# Impact of Novel Resistance Profiles in HIV-1 Reverse Transcriptase on Phenotypic Resistance to NVP

**DOI:** 10.1155/2012/637263

**Published:** 2012-03-21

**Authors:** Liyan Jiao, Hanping Li, Lin Li, Daomin Zhuang, Yongjian Liu, Zuoyi Bao, Siyang Liu, Jingyun Li

**Affiliations:** State Key Laboratory of Pathogen and Biosecurity, Institute of Microbiology and Epidemiology, Academy of Military Medical Science, Beijing 100071, China

## Abstract

*Objective*. To clarify the impact of H221Y mutation on drug resistance to NVP. *Methods*. 646 bp HIV-1 *pol* gene fragments (from 592 to 1237 nucleotide) with different NNRTIs mutation profiles from AIDS patients receiving antiretroviral therapy containing NVP regimens were introduced into pNL4-3 backbone plasmid. H221Y and (or) Y181C mutations were reverted to wild type amino acids by site-directed mutagenesis, then strains containing various mutation patterns were packaged. Phenotypic drug resistance was analyzed on TZM-bl cells. *Results*. 12 strains containing different drug-resistant mutation profiles were constructed, including the K101Q series (K101Q/Y181C/H221Y, K101Q/Y181C, K101Q/H221Y, and K101Q), the V179D series (V179D/Y181C/H221Y, V179D/Y181C, V179D/H221Y, and V179D), and the K103N series (K103N/Y181C/H221Y, K103N/Y181C, K103N/H221Y, K103N). For strains containing the mutation profiles (K101Q/Y181C, K101Q, V179D/Y181C, V179D, K103N/Y181C, and K103N), the presence of H221Y reduced NVP susceptibility by 2.1 ± 0.5 to 3.6 ± 0.5 fold. To the mutation profiles K101Q/H221Y, K101Q, V179D/H221Y, V179D, K103N/H221Y, and K103N, the presence of Y181C reduced NVP susceptibility by 41.9 ± 8.4 to 1297.0 ± 289.1 fold. For the strains containing K101Q, V179D, and K103N, the presence of Y181C/H221Y combination decreased NVP susceptibility by 100.6 ± 32.5 to 3444.6 ± 834.5 fold. *Conclusion*. On the bases of various NNRTIs mutation profiles, Y181C remarkably improved the IC_50_ to NVP, although H221Ymutation alone just increases 2.1 *∼* 3.6-fold resistance to NVP, the mutation could improve 100.6 *∼* 3444.6-fold resistance to NVP when it copresent with Y181C, the phenotypic drug resistance fold was improved extremely. For strains containing the mutation profiles (K101Q/Y181C, K101Q, V179D/Y181C, V179D, K103N/Y181C, and K103N), the presence of H221Y reduced NVP susceptibility by 2.1 ± 0.5 to 3.6 ± 0.5 fold.

## 1. Introduction

 The nonnucleoside reverse transcriptase inhibitors (NNRTIs) are small molecules which can bind to a nonactive hydrophobic site pocket close to the catalytic domain of RT (termed NNRTI-binding pocket). The binding of NNRTIs to RT could alter the structure of RT and block the polymerase activity of RT. As the results, the replications of HIV-1 were inhibited. However, despite their high efficiencies and low toxicities, the use of NNRTIs is hampered owing to the rapid selection of drug-resistant HIV-1 strains [1, 2]. Therefore, a deeper understanding of resistance is necessary in order to effectively prevent and manage HIV-1 drug resistance.

 Almost all NNRTIs-related mutations were identified with their location in binding pocket or near the pocket [3]. These mutations can change the size, shape, and polarity of the pocket and affect the binding of NNRTIs accordingly [4]. There are four types of NNRTIs-resistant mutations: (1) Primary mutation: these mutations emerge earliest during therapy and cause high-level resistance to one or more NNRTIs, including K103N/S, Y181C/I/V, V106A/M, Y188L/C/H, and G190A/S/E. All these mutations can lead to high-level resistance to NVP. (2) Secondary mutation: these mutations usually appear with primary mutations but occasionally occur by themselves. Several of these mutations, particularly K101E/P and M230L, have been associated with significant resistance to multiple NNRTIs. (3) Minor nonpolymorphic mutation and accessory mutation: these mutations emerge alone or couple with other NNRTIs-resistant mutations and confer decreasing NNRTIs susceptibility persistently. This type of mutation consists of A98G, K101Q, V108I, and V179D/E. These mutations, such as K101Q and V179I, often occur with other primary mutations. In previous study, in contrast with NRTIs resistance, a single NNRTIs mutation in HIV-1 RT can lead to high-level resistance to multi-NNRTIs (including efavirenz and nevirapine). So in the past, researchers seldom pay attention to the NNRTIs-resistant profiles resulting from therapy failure because a single NNRTI treatment failure always means the second NNRTI therapy failure [5–7].

 H221Y was recently found as an NNRTIs mutation which confers resistance to NVP. Our previous study showed that the virus carrying K103N/Y181C/H221Y mutation combination could replicate stably in vitro in absence of drugs [8]. However, the impact of H221Y on NNRTI susceptibility has been poorly characterized, and the interaction of H221Y with other mutations is yet indistinct. The objective of this study is to clarify the impact of H221Y by itself and combination with other mutations on drug resistance to NVP.

## 2. Materials and Methods

**Selection of Plasma Samples**

Plasma samples were collected from 3 AIDS patients in clinical cohort receiving antiretroviral therapy [nevirapine (NVP) + zidovudine (AZT) + didanosine (ddI)]. All patients have written informed consent. Genotype-resistant results of these patients showed all samples contained three NNRTIs mutations (Y181C/H221Y plus another mutation) which conferred resistance to NVP.

**Viral RNA Extraction and One-Step RT PCR**

RNA was extracted from 200 *μ*L plasma using High Pure Viral RNA Kit (Roche Corp.) following the protocol provided with kits. HIV-1 pol protease and reverse transcriptase genes were obtained by one-step RT PCR.

**Cloning of the HIV-1 Protease and RT (Reverse Transcriptase) Gene PCR Products**

The PCR products were cleaned by Wizard SV Gel and PCR Clean-Up System (Promega Corp.) and ligated with PMD18-T Vector (TaKaRa Biotechnology Co., Ltd.), then transformed into *E. coli* DH5*α*.

**Selection of Clones with 3 Different Mutation Profiles to NVP**

Several clones from each transformation were sequenced, and 3 clones containing 3 different NNRTIs mutation profiles, including K101Q/Y181C/H221Y, V179D/Y181C/H221Y, and K103N/Y181C/H221Y, were selected.

**Construction of 12 Clones with Different NVP-Resistant Mutation Profiles Using Site-Directed Mutagenesis**

Three fragments were constructed to convert mutant RT 221 (Y) to wild-type (H) or (/and) convert mutant RT 181 (C) to wild-type (Y) by site-directed mutant-genesis kit (TaKaRa Mutan BEST kit, TaKaRa Biotechnology Co. Ltd.) with mutagenic primers: 221Y→H, 5′-ACAAAAAACATCAGAAA GAACCTCCAT-3′ (H221-F (C-6/P-110)), 5′-ACAAAAAACATCAGAAAGAACCCCC-3′ (H221-F(P-22)), 5′-CTGGTGTGGTAAATCCCCACCT-3′ (H221-R(C-6-2)), 5′-CTGGTGTGTAAAACCCCCACCTTAAC-3′ (H221-R (P-22)), and 5′-CTGGTGT GTAAAATCCCCACCTCAA-3′ (H221-R(P-110)); 181C→Y, 5′-ATAGTTATC TATC AATACATGGATGATTTGTATGT-3′ (Y181-F(C-6/P-110)829), 5′-ATGGAAATCT ATCAATACATGGATGATTTGT-3′ (Y181-F(P-22-6)829), 5′-GTCTGGATTTTGTTTTCTAAAAGGCTC-3′ (Y181-R 828). Different mutation profiles were created including K101Q/Y181C/H221Y, K101Q/Y181C, K101Q/H221Y, K101Q, V179D/Y181C/H221Y, V179D/Y181C, V179D/H221Y, V179D, K103N/Y181C/H221Y, K103N/Y181C, K103N/H221Y, and K103N. All of mutations were confirmed by sequencing with primers: 5′-GCCATAAAGAAAAAARACAGTACTARA-3′ (Mut-F 481) and 5′-TCATTCTTGCATAYTTTCCTGT TTT-3′ (Mut-R 1369).

**Insertion of the RT Fragments into the Backbone pNL4-3 Vector**

Two restriction sites (*Sbf/* and *Age/*) were created in the 12 fragments with different mutation profiles. The 646 bp fragments were digested with *Sbf/* and *Age/*, purified with Wizard SV Gel and PCR Clean-Up System (Promega Corp.), and inserted into backbone pNL4-3 vector which had been removed the corresponding fragments.

**Preparation of Plasmid DNA**

The mutations introduced into pNL4-3 vectors were confirmed by sequencing primers: 5′-GCCATAAAGAAAAAAGACAGTACTAAA-3′ (NL + aim-F481) and 5′-CCTTCATTCTTGCATATTTTCCT-3′ (NL + aim-R 1372) before being prepared by QIAGEN Plasmid Midi Kit (25) (QIAGEN Corp.).

**Cells and Media**

The 293T and the TZM-bl cell lines were presented by the nucleic acid vaccine laboratory of University of Massachusetts Medical School and were maintained in DMEM (Invitrogen, Corp.) medium containing 10% fetal bovine serum.

**Transfections, Infections, and NVP Susceptibility Assays**

The ligated pNL4-3 plasmids were transfected into 293T cells by using Lipofectamine 2000 (Invitrogen, Corp.). TCID_50_ of the virus culture supernatant and IC_50_s to NVP were detected by using TZM-bl cells. The IC_50_s were determined by concentration-response curves of the log_10_ NVP concentration versus percent inhibition of virus.

## 3. Results

**Construction 12 HIV-1 Virus Containing Different Mutation Profiles**

These viruses were named 101Q-1, 101Q-2, 101Q-3, 101Q-4, 179D-1, 179D-2, 179D-3, 179D-4, 103N-1, 103N-2, 103N-3, and 103N-4; the mutation patterns they contained were K101Q/Y181C/H221Y, K101Q/Y181C, K101Q/H221Y, K101Q, V179D/Y181C/H221Y, V179D/Y181C, V179D/H221Y, V179D, K103N/Y181C/H221Y, K103N/Y181C, K103N/H221Y, and K103N. As the control virus, Con-1 was pNL4-3 without any drug-resistant mutations. The virus name and corresponding drug resistance mutation profiles were indicated at Table 1.

**IC_50_s of 13 Virus**

Firstly, we measured the TCID_50_s of recombinant virus with TZM-bl cells. TCID_50_s of virus 101Q-1, 101Q-2, 101Q-3, 101Q-4, 179D-1, 179D-2, 179D-3, 179D-4, 103N-1, 103N-2, 103N-3, 103N-4, and Con-1 were 10687, 6250, 18275, 6250, 156250, 21555, 45305, 31250, 31250, 18275, 13975, and 4780 per milliliter, respectively. To determine the IC_50_s of NVP versus every virus, we identified the concentration range of high and low inhibition and ensured that the greatest concentration can inhibit 95% viruses at least, and the lowest concentration can inhibit 10 ~ 15%. NVP concentrations of all viruses were diluted double successively except virus Con-1 triplicate successively. The greatest concentrations of every virus were showed in Table 1.

**Figure 1:**
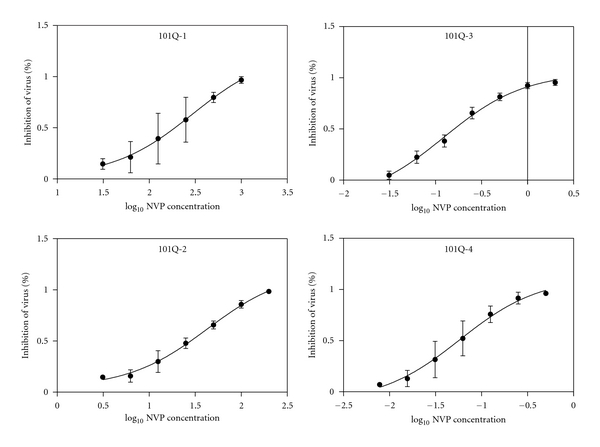
The curves of percent inhibition of virus 101Q-1~101Q-4 to log_10_ NVP concentration.

**Figure 2:**
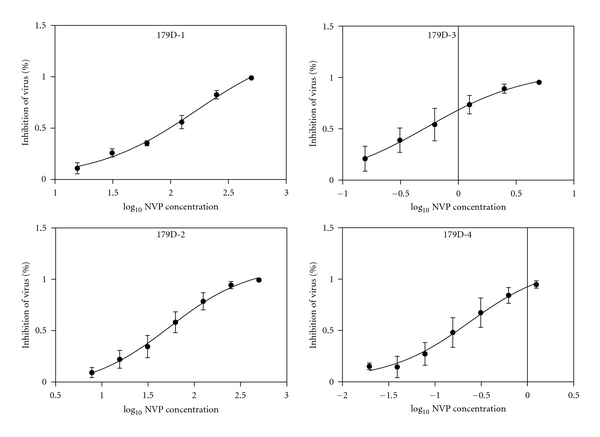
The curves of percent inhibition of virus 179D-1~179D-4 to log_10_ NVP concentration.

**Figure 3:**
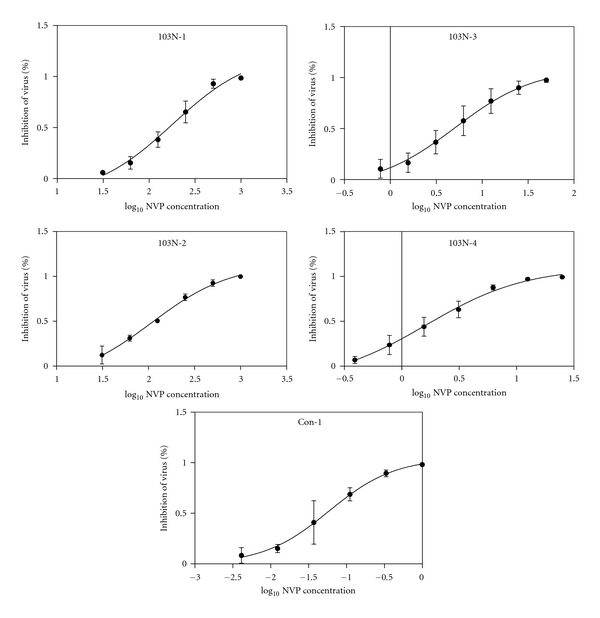
The curves of percent inhibition of virus 103N-1~103N-4, Con-1 to log_10_ NVP concentration.

Three independent experiments were performed in triplicate. The curves of percent inhibition of virus to log_10_ NVP concentration were created and showed in Figures 1, 2 and 3. IC_50_s of 13 viruses were separately 108.2 ± 1.9 *μ*M, 47.8 ± 18.1 *μ*M, 0.14 ± 0.05 *μ*M, 0.04 ± 0.01 *μ*M, 162.6 ± 47.5 *μ*M, 45.0 ± 12.8 *μ*M, 0.4 ± 0.0 *μ*M, 0.1 ± 0.0 *μ*M, 191.1 ± 91.4 *μ*M, 89.2 ± 22.7 *μ*M, 4.4 ± 0.9 *μ*M, 2.0 ± 0.4 *μ*M, and 0.03 ± 0.01 *μ*M (Table 1).

**Susceptibility Reduction of Various Mutation Patterns Compared to pNL4-3**

Compared to pNL4-3, the resistance values (folds of IC_50_ increased) derived from viruses carrying different mutation profiles such as K101Q/Y181C/H221Y, K101Q/Y181C, K101Q/H221Y, K101Q, V179D/Y181C/H221Y, V179D/Y181C, V179D/H221Y, V179D, K103N/Y181C/H221Y, K103N/Y181C, K103N/H221Y, and K103N were 3740.8 ± 314.1, 1490.9 ± 354.4, 4.4 ± 1.2, 1.2 ± 0.2, 5090.6 ± 744.6, 1413.0 ± 269.8, 13.4 ± 1.9, 4.6 ± 1.3, 5859.3 ± 1648.2, 2802.4 ± 124.1, 139.2 ± 8.2, and 63.8 ± 3.1 folds, respectively (Table 1).

**Association of H221Y with an Increased NVP IC_50_ Based on Various Mutation Profiles**

As for different mutation patterns including K101Q/Y181C, K101Q, V179D/Y181C, V179D, K103N/Y181C and K103N, H221Y conferred NVP IC_50_ increased folds 2.2 ± 0.9, 3.2 ± 0.3, 3.6 ± 0.5, 3.0 ± 0.4, 2.1 ± 0.5, and 2.2 ± 0.1, respectively (Table 2).

**Association of Y181C with an Increased NVP IC_50_ Based on Various Mutation Profiles**

In relation to various mutation profiles such as K101Q/H221Y, K101Q, V179D/H221Y, V179D, K103N/H221Y, and K103N, Y181C mutation increased folds of NVP and IC_50_s were 759.1 ± 41.9, 1297.0 ± 289.1, 390.0 ± 101.6, 312.5 ± 45.3, 41.9 ± 8.4, and 47.9 ± 4.2, respectively (Table 2).

**Association of Y181C/H221Y with an Increased NVP IC_50_ Based on Various Mutation Profiles**

Concerning K101Q, V179D, and K103N, Y181C/H221Y mutation combination improved the IC_50_s of NVP 3444.6 ± 834.5, 1132.6 ± 180.4, and 100.6 ± 32.5 folds, respectively (Table 2).

**The Complete Set of Differences from Consensus B**

The differences from consensus B were listed in Table 3, and these additional mutations might affect susceptibility.

## 4. Discussion

 H221Y was first reported related to NRTI treatment in 2003 [9], but in recent study, H221Y was concerned associated with exposed to NNRTIs [6, 10]. Ceccherini-Silberstein et al. found the prevalence rate of H221Y in isolates from patients failing NVP treatment was 10.3% and the mutation was included in the top 10 and 15 determinants for NVP and EFV resistance, respectively, ranking even above some classical NNRTI resistance mutations, such as K101E, V108I, and G190E [6]. The group found H221Y was strongly associated with the use of NVP and showed positive interactions with Y181C and was also negatively associated with the use of ZDV and with TAMs (particularly TAMs-2, such as D67N, K70R, K219Q/E, and T215F) and was then associated with an increased susceptibility to ZDV. When H221Y was copresent with TAMs-2, the ZDV susceptibility was even greater than that observed when TAMs-2 were copresent with Y181C. The presence of H221Y along with Y181C was associated with a 12.4-fold increase in NVP resistance. Jiang et al. found that the occurrence of the H221Y was 19.8% in 91 patients receiving nevirapine and lamivudine plus stavudine (57.1%) or zidovudine (42.9%) [10]. Reuman et al. analyzed viruses from 13039 individuals with sequences containing at least one of 52 published NNRTI-selected mutations; the frequency of H221Y among 1510 viruses from individuals who received nevirapine but no other NNRTI was 12%. H221Y occurred along with Y181C in our study (data not shown) and the virus with mutation pattern K103N/Y181C/H221Y can replicate stably in vitro without drug pressure [8]. Our study showed that as for the mutation profiles K101Q/Y181C, K101Q, V179D/Y181C, V179D, K103N/Y181C, and K103N, the presence of H221Y, respectively lead to 2.2 ± 0.9, 3.2 ± 0.3, 3.6 ± 0.5, 3.0 ± 0.4, 2.1 ± 0.5, and 2.2 ± 0.1-fold increase in NVP resistance.

 Y181C is a very important NNRTIs-related drug resistant mutation, and the mutation can cause high-level resistance to NVP and DLV and low-level resistance to EFV; otherwise, this mutation can increase susceptibility to AZT and TDF. Sungkanuparph group reported the prevalence rate of Y181C was 59.5% among the 158 NNRTI failure patients (for a median NNRTI treatment period of 88 weeks) [11]. Taiwo et al. found the frequency of Y181C was 42.9% in the patients receiving NNRTI treatment for a median period of 53 weeks [12]. Y181C presents in 40% of patients failing NVP and has the third-greatest weight in the SVR (support vector regression) model for NVP resistance [6]. In Reuman group's study [13], Y181C is the most common resistant mutation among the sequences from patients receiving NVP (48%), and nearly 17% (*n* = 2233) sequences contained three or more NNRTIs-resistant mutations and these NNRTIs-resistant profiles often carried Y181C and occurred with one or more thymidine analogue mutations, which suggested these mutation pattern might significantly associated with the function of HIV-1 RT. Many studies have reported the phenotype resistance of Y181C, Bacheler et al. [14] found the virus with K103N/Y181C and other mutations conferring above 1600-fold resistance to NVP, and Qari et al. [15] covered the virus containing K101E/Y181C/G190A and other mutations could increase 893-fold resistance to NVP. As we have known [16], Y181C copresent with TAMs2 could increase the susceptibility to ZDV. In our study, mutation patterns K101Q/H221Y, K101Q, V179D/H221Y, V179D, K103N/H221Y, and K103N, Y181C could, respectively, improve 759.1 ± 41.9, 1297.0 ± 289.1, 390.0 ± 101.6, 312.5 ± 45.3, 41.9 ± 8.4, and 47.9 ± 4.2-fold to NVP resistance.

 Although H221Y mutation alone just increases 2.1 ~ 3.6-fold resistance to NVP, the mutation could improve 100.6 ~ 3444.6-fold resistance to NVP when it copresent with Y181C. To be specific, to K101Q, V179D, and K103N, Y181C/H221Y could confer 3444.6 ± 834.5, 1132.6 ± 180.4, and 100.6 ± 32.5-fold resistance to NVP.

 Ceccherini-Silberstein et al. [6] described the resistant characters of 9 novel NNRTI-related mutations including K101Q, I135M/T, H221Y, K223E/Q, L228H/R, and V179I. In these novel mutations, K101Q and I135T copresented with K103N which related to the increase resistance of EFV and NVP. Residue 101 may establish hydrogen bonds with EFV and may directly interact with correlated residue 102 by van der Waals interaction. In previous study, the appearance of K101Q might be based on the occurrence of K103N [17]. In other study [18], residue 101 showed negative association with HLA-A2 genotype which suggested, in the presence of HLA-A2 restricted immune response, position 101 may be under negative selective pressure that favors the preservation of the wild-type amino acid. As for residue 179, only V179F mutation did not decrease the susceptibility to etravirine; however, when the mutation combined with Y181C, the viral etravirine susceptibility could be reduced more than 100-fold [19–22]. Thus receiving NNRTIs treatment on long term might result in the accumulation of accessory mutations and lead to higher level of drug resistance.

 With the upcoming approval of the new NNRTIs, understanding the efficacy of these NNRTIs mutation profiles shows more and more importance. Further in vitro and clinical studies are therefore necessary to confirm the efficacy of these new mutation patterns. These studies will provide information for deeper understanding of the mechanisms of drug resistance.

## 5. Conclusions

 The success of HAART (Highly Active Antiretroviral Therapy) in treating AIDS patients is hampered by the emergence of drug-resistant strains, and more and more studies indicate that there are more drug-resistant mutations than we have known. The roles of these novel mutations in drug resistance have still been indistinct. In this study, we found that Y181C remarkably improved the IC_50_ to NVP, although the novel mutation H221Y only slightly conferred resistance to NVP, when it combined with Y181C, the phenotypic drug resistance folds were improved extremely.

## Figures and Tables

**Table 1:** The greatest initial NVP concentrations of every virus, IC_50_ (*μ*M) of every virus and the increased NVP resistance compared to pNL4-3.

Virus no.	Mutation patterns	Greatest concentration of NVP (*μ*M)	IC_50_ (*μ*M)	Median fold of IC_50_ increased compared to pNL4-3 (±S.D.)
101Q-1	K101Q/Y181C/H221Y	1000	108.2 ± 1.9	3740.8 (±314.1)
101Q-2	K101Q/Y181C	200	47.8 ± 18.1	1490.9 (±354.4)
101Q-3	K101Q/H221Y	2	0.14 ± 0.05	4.4 (±1.2)
101Q-4	K101Q	0.5	0.04 ± 0.01	1.2 (±0.2)
179D-1	V179D/Y181C/H221Y	500	162.6 ± 47.5	5090.6 (±744.6)
179D-2	V179D/Y181C	500	45.0 ± 12.8	1413.0 (±269.8)
179D-3	V179D/H221Y	5	0.4 ± 0.0	13.4 (±1.9)
179D-4	V179D	1.25	0.1 ± 0.0	4.6 (±1.3)
103N-1	K103N/Y181C/H221Y	1000	191.1 ± 91.4	5859.3 (±1648.2)
103N-2	K103N/Y181C	1000	89.2 ± 22.7	2802.4 (±124.1)
103N-3	K103N/H221Y	50	4.4 ± 0.9	139.2 (±8.2)
103N-4	K103N	25	2.0 ± 0.4	63.8 (±3.1)
Con-1		1	0.03 ± 0.01	

**Table 2:** Contribution of H221Y, Y181C, H221Y/Y181C to an increased NVP resistance based on various mutation profiles.

Aimed mutation	Base mutation profile	Increased fold of IC_50_ (±S.D.)
H221Y	K101Q/Y181C	2.2 (±0.9)
H221Y	K101Q	3.2 (±0.3)
H221Y	V179D/Y181C	3.6 (±0.5)
H221Y	V179D	3.0 (±0.4)
H221Y	K103N/Y181C	2.1 (±0.5)
H221Y	K103N	2.2 (±0.1)
Y181C	K101Q/H221Y	759.1 (±41.9)
Y181C	K101Q	1297.0 (±289.1)
Y181C	V179D/H221Y	390.0 (±101.6)
Y181C	V179D	312.5 (±45.3)
Y181C	K103N/H221Y	41.9 (±8.4)
Y181C	K103N	47.9 (±4.2)
Y181C/H221Y	K101Q	3444.6 (±834.5)
Y181C/H221Y	V179D	1132.6 (±180.4)
Y181C/H221Y	K103N	100.6 (±32.5)

**Table 3:** The complete set of differences from consensus B (from 99 to 313 AA in pol region).

Virus	Position of AA in pol region																				
	**101**	103	135	162	178	179	**181**	200	203	207	**215**	**221**	228	272	277	278	292	293	297	311	312
Consensus B	**K**	K	I	S	I	V	**Y**	T	E	Q	**T**	**H**	L	A	K	Q	V	I	E	K	E
101Q-1	**Q**	—	R	C	—	—	**C**	A	—	—	—	**Y**	—	—	R	E	—	—	—	—	—
101Q-2	**Q**	—	R	C	—	—	**C**	A	—	—	—	—	—	—	R	E	—	—	—	—	—
101Q-3	**Q**	—	R	C	—	—	—	A	—	—	—	**Y**	—	—	R	E	—	—	—	—	—
101Q-4	**Q**	—	R	C	—	—	—	A	—	—	—	—	—	—	R	E	—	—	—	—	—
179D-1	—	—	V	C	M	D	**C**	A	G	E	**Y**	**Y**	R	—	R	E	I	V	—	—	—
179D-2	—	—	V	C	M	D	**C**	A	G	E	**Y**	—	R	—	R	E	I	V	—	—	—
179D-3	—	—	V	C	M	D	—	A	G	E	**Y**	**Y**	R	—	R	E	I	V	—	—	—
179D-4	—	—	V	C	M	D	—	A	G	E	**Y**	–	R	—	R	E	I	V	—	—	—
103N-1	—	N	V	C	—	—	**C**	A	—	—	**Y**	**Y**	—	S	R	E	—	—	K	S	K
103N-2	—	N	V	C	—	—	**C**	A	—	—	**Y**	—	—	S	R	E	—	—	K	S	K
103N-3	—	N	V	C	—	—	—	A	—	—	**Y**	**Y**	—	S	R	E	—	—	K	S	K
103N-4	—	N	V	C	—	—	—	A	—	—	**Y**	—	—	S	R	E	—	—	K	S	K
